# Anti-Inflammatory Applications of Melittin, a Major Component of Bee Venom: Detailed Mechanism of Action and Adverse Effects

**DOI:** 10.3390/molecules21050616

**Published:** 2016-05-11

**Authors:** Gihyun Lee, Hyunsu Bae

**Affiliations:** Department of Physiology, College of Korean Medicine, Kyung Hee University, 1 Hoeki-Dong, Dongdaemoon-gu, Seoul 130-701, Korea; glee@khu.ac.kr

**Keywords:** bee venom, melittin, inflammation

## Abstract

Inflammation is a pervasive phenomenon triggered by the innate and adaptive immune systems to maintain homeostasis. The phenomenon normally leads to recovery from infection and healing, but when not properly phased, inflammation may cause immune disorders. Bee venom is a toxin that bees use for their protection from enemies. However, for centuries it has been used in the Orient as an anti-inflammatory medicine for the treatment of chronic inflammatory diseases. Bee venom and its major component, melittin, are potential means of reducing excessive immune responses and provide new alternatives for the control of inflammatory diseases. Recent experimental studies show that the biological functions of melittin could be applied for therapeutic use *in vitro* and *in vivo*. Reports verifying the therapeutic effects of melittin are accumulating in the literature, but the cellular mechanism(s) of the anti-inflammatory effects of melittin are not fully elucidated. In the present study, we review the current knowledge on the therapeutic effects of melittin and its detailed mechanisms of action against several inflammatory diseases including skin inflammation, neuroinflammation, atherosclerosis, arthritis and liver inflammation, its adverse effects as well as future prospects regarding the use of melittin.

## 1. Introduction

Inflammation is a primary process of the immune response that is triggered by any stimulus such as infection, injury, and exposure to contaminants that poses a real or perceived threat to homeostasis [1]. It is a protective process for the body, however chronic inflammation can cause the development of different diseases like rheumatoid arthritis, cardiovascular disease, diabetes, obesity, inflammatory bowel disease, asthma, and CNS related diseases such as Parkinson’s disease and Amyotrophic Lateral Sclerosis (ALS) [2].

**Figure 1:**
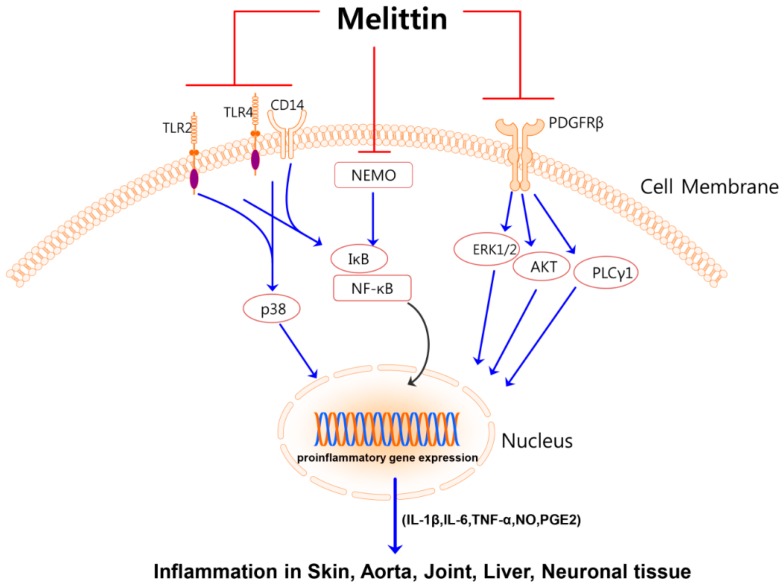
Major mechanisms for the anti-inflammatory activities of melittin. Melittin suppresses signal pathways of TLR2, TLR4, CD14, NEMO, and PDGFRβ. By inhibiting these pathways melittin decreases activation of p38, ERK1/2, AKT, PLCγ1 as well as translocation of NF-κB into the nucleus. This inhibition results in reduced inflammation in skin, arota, joint, liver, and neuronal tissue. TLR, toll-like receptor; CD, cluster of differentiation; NEMO, nuclear factor kappa-B essential modulator; PDGFRβ, Platelet-derived growth factor receptor beta.

While bee venom is a toxin which bees use for their protection from enemies, a number of recent studies regarding the beneficial roles of bee venom report that it possesses radioprotective [3], anti-mutagenic [4], anti-nociceptive [5,6,7], anti-cancer [8,9,10,11,12,13,14] and anti-inflammatory [15,16,17,18,19,20] activities. Melittin is the principal constituent of bee (*Apis mellifera*) venom, accounting for approximately 50% by weight of dried bee venom [21]. This amphiphilic peptide has a linear structure consists of 26 amino acids (NH_2_-GIGAVLKVLTTGLPALISWIKRKRQQ-CONH_2_) [22]. In high doses, melittin may cause itching, inflammation, and local pain; on the other hand, small doses of melittin produce broad anti-inflammatory effects [23]. There are several reviews available in the literature that focus on diverse functions as well as the pharmacological aspects of melittin [23,24,25,26,27,28,29,30,31,32]. However, reviews of the anti-inflammatory role of melittin alone are currently not available. Numerous recent reports point to several anti-inflammatory mechanisms of melittin in different types of disease models. Herein, we highlight the newest findings on the beneficial role and mechanisms of melittin against inflammatory disorders (Table 1, Figure 1). We also summarize possible adverse effects of melittin and attempts to overcome them.

## 2. Therapeutic Applications of the Anti-Inflammatory Effects of Melittin

### 2.1. Application for Skin Inflammation

Acne vulgaris is a long term skin disorder of the hair follicle in the face and upper trunk, and *Propionibacterium* *acnes* (*P. acnes*) is the main cause of the inflammation of acne [33]. Toll-like receptor 2 signaling activated by *P. acnes* causes keratinocytes and monocytes to secrete pro-inflammatory cytokines, including TNF-α, IL-1β, and IL-8 [34,35]. A wide range of agents, including antibiotics, is used to suppress inflammation in acne vulgaris, but antibiotics can cause side effects [36].

The protective effects of melittin on *P. acnes*-induced inflammatory responses *in vitro* and *in vivo* were reported by Lee *et al.* [37]. They investigated the anti-inflammatory effects of melittin treatment in heat-killed *P. acnes*-treated HaCaT cells. Melittin treatment attenuated the increased phosphorylation of IKK, IκB, NF-κB as well as p38 by heat-killed *P. acnes* in HaCaT cells. These data show that melittin treatment abrogates *P. acnes*-induced inflammatory cytokine production through blocking NF-κB signaling as well as p38 MAPK signaling in HaCaT cells. In addition, the anti-inflammatory effect of melittin was examined in a live *P. acnes*-induced inflammatory skin disease animal model. In the animal model, melittin-treated ears show markedly reduced *P. acnes*-injected swelling and granulomatous responses, as compared with ears injected with live *P. acnes* alone.

Another study showed the inhibitory action of melittin against heat-killed *P. acnes*-induced apoptosis and inflammation in human THP-1 monocytic cells [38]. Melittin treatment suppressed the cleavage of the caspase-3, -8, and PARP in heat-killed *P. acnes*-treated THP-1 monocytic cells; in addition, administration of melittin significantly decreased the expression of TNF-α and IL-1β.

### 2.2. Application for Neurodegenerative Diseases

*In vitro* assays have revealed the potential of melittin as an agent for the prevention of neurodegenerative diseases. Moon *et al.* showed that melittin has a potent suppressive effect on pro-inflammatory responses of BV2 microglia and suggested that melittin may have potential to treat neurodegenerative diseases accompanied with microglial activation [39]. Melittin suppresses expression of NO and iNOS by blocking LPS-induced activation of NF-κB in BV2 microglial cell line. These results indicate that melittin suppresses COX-2/PGE2 expression resulting in anti-inflammatory properties. Meanwhile, Han *et al.* investigated the anti-apoptotic effects of melittin in a H_2_O_2_-induced cytotoxicity model using the SH-SY5Y human neuroblastoma cell line [40]. Melittin treatment increased cell viability and decreased apoptotic DNA fragmentation. Melittin inhibited the H_2_O_2_-induced decrease of anti-apoptotic factor Bcl-2 expression and increase of pro-apoptotic factor Bax expression.

Amyotrophic lateral sclerosis (ALS) is a progressive neurodegenerative disorder that affects motor neurons in the brain and the spinal cord, resulting in weakness and atrophy of muscles [53]. Yang *et al.* showed that melittin treatment (0.1 μg/g twice a week s.c. acupuncture on bilateral point ST36) improves anti-neuroinflammatory ability of proteasome in the CNS of ALS model mice [41]. In this model, administration of melittin reduces phosphorylation of p38 and the microglial cell number in the brainstem and spinal cord; in addition, melittin-treated mice exhibit declined neuronal death in the spinal cord and improved motor control that improves motor function. Furthermore, melittin alleviates the misfolding of proteins by activation of chaperones and reducing post-transcriptional modification of α-synuclein, a major process of ALS pathogenesis. The results demonstrate the anti-neuroinflammatory effects of melittin. Although the main problem of ALS occurs in CNS, ALS also affects other organs, including the liver, spleen, and lung. Melittin treatment attenuates inflammation and stimulates the signaling for cell survival in the spleen and lung in an animal model of ALS [42]. Administration of melittin suppresses the expression of CD14 and Iba-1 (inflammatory proteins) in the lung of symptomatic ALS transgenic mice. In addition, melittin increases the expression of pERK and Bcl-2 (cell survival factors) and suppresses the expression of C14 and COX-2 in the spleen of symptomatic ALS mice. 

Dantas *et al.* examined the pharmacological effects of melittin in mice, with particular emphasis on dopaminergic related behaviors [54]. The animals were submitted to behavioral tests, such as the apomorphine rotation test, catalepsy test, and open field test. The results showed that melittin-treated mice displayed reduced effects caused by apomorphine, though there is no alteration in motor activity or cataleptic effects with melittin treatment. The authors reported that melittin exhibits anti-psychotic properties and could be an alternative to treat psychotic disorders, cutting down the side effects of neuroleptic medicines. Melittin also decreases apomorphine-induced stereotypes. The data indicate the anti-psychotic activity of melittin in a mice model.

### 2.3. Application for Atherosclerosis

Atherosclerosis is an inflammatory disease in which plaque builds up inside arteries. This chronic inflammatory disorder of the arteries is a one of major causes of death in adults. Plaque is made up of cholesterol, triglycerides, remnants of dead cells, and immune cells. In the plaque, inflammatory immune cells including macrophage and helper T cells produce inflammatory cytokines, a main trigger of plaque growth [55]. Modulation of NF-κB signaling, which plays a critical role in apoptosis and cell proliferation, is regarded as a potential therapeutic target for the treatment of atherosclerosis [56,57].

*In vitro* studies show an effect of melittin on the proliferation and apoptosis of vascular smooth muscle cells (VSMCs). The NF-κB signal pathway was investigated as a potent mechanism for apoptosis of cultured rat aortic VSMCa. Melittin suppresses not only platelet-derived growth factor receptor (PDGFR) β-tyrosine phosphorylation, but also downstream intracellular signal transduction in rat aortic VSMCa [43] and blocks phosphorylation of AKT induced by the PDGRF signal [44]. In addition, phosphorylation of extracellular signal-regulated kinase 1/2, an upstream signal of NF-κB, is slightly suppressed by melittin treatment. Melittin treatment, besides, induces expression of pro-apoptotic proteins, including p53, Bax, and caspase-3, but decreases expression of anti-apoptotic protein Bcl-2 [44]. In addition, *in vivo* experiments demonstrate that melittin treatment suppresses atherosclerosis via athero-protective functions in High-Fat/LPS treated mice [45]. Melittin treatment decreases total cholesterol and triglyceride levels and recovers heart and descending aorta. In addition, melittin decreases the expression levels of TNF-α, IL-1β, VCAM-1, ICAM-1, and TGFβ-1 in atherosclerotic mice.

Cho *et al.* used mass spectrometry and gel electrophoresis to elucidate the anti-atherosclerotic mechanisms of melittin. They identified differentially expressed proteins by melittin treatment in human VSMCs stimulated by TNF-α [46]. In their experiments, a proteomics analysis identify 33 proteins with consistently different expression patterns after melittin treatment. The identified proteins include anti-apoptotic proteins like as stress-70 protein and annexin A1, Several target molecules of inflammation and proliferation such as prohibitin, annexin-1, and calreticulin were identified. Furthermore, intracellular signal pathway analysis shows that EGFR and NF-κB are the main signaling molecules of inflammation in TNF-α treated human VSMCs.

### 2.4. Application for Arthritis

The Hong group proposed target inactivation of NF-κB by directly binding to the p50 subunit as anti-arthritic mechanism of bee venom and melittin. They demonstrated that melittin inhibits LPS-induced p50 translocation into nucleus resulting in reduced transcription of inflammatory genes [20]. Melittin directly binds to p50 (affinity [Kd] = 1.2 × 10^−8^ M). They also investigated the anti-inflammatory effect of melittin via interaction with IKKs. In a mouse macrophage cell line and synoviocytes acquired from rheumatoid patients, melittin suppresses the TNF-α/LPS-induced production of NO and PGE2 [47]. In addition, they demonstrated that the JNK pathway is involved in the inhibition of inflammatory target gene expression and NF-κB activation by melittin [48].

### 2.5. Application for Liver Inflammation

Park *et al.* reported that melittin attenuates hepatic injury, inflammation and hepatic fibrosis. Melittin inhibits TNF-α secretion and expression of IL-1β and IL-6 in the TNF-α-treated hepatic stellate cells (HSCs). Melittin attenuates inflammation and fibrosis by inhibiting the NF-κB signaling pathway in thioacetamide-induced liver fibrosis. In addition, they suggested that the regulated inflammatory response may affect anti-fibrotic effect of melittin in the activated HSCs [49]. Subsequently, they demonstrate that melittin inhibits liver failure via blocking NF-κB signaling and apoptotic pathways in the D-galactosamine/LPS-induced mouse liver failure model [50]. 

Melittin decreases the high rate of lethality, alleviates hepatic pathological injury, attenuates hepatic inflammatory responses and inhibits hepatocyte apoptosis. They also elucidated the inhibitory mechanism of melittin for NF-κB transcription in TNF-α-induced hepatic damage [51]. In TNF-α-treated hepatocytes, melittin inhibits DNA binding of NF-κB as well as promoter activity of NF-κB, suggesting that melittin inhibits apoptosis of hepatocytes through suppression of NF-κB activation. Recently, they showed that administration of melittin attenuated inflammation and fibrosis of the bile duct. Their experimental results suggest that melittin may have therapeutic applications in chronic liver injury [52]. Figure 1 summarizes the major mechanisms of the anti-inflammatory action of melittin.

## 3. Adverse Effects of Melittin

Melittin is the major constituent of apitoxin and is known as an allergenic peptide. It is also responsible for cell lysis and death. Accumulated melittin peptides disrupt phospholipid packing in the cell membrane, resulting in cell lysis [23]. Melittin provokes the lysis of plasma membranes and intracellular membranes. Melittin and bee venom phospholipase A_2_ (PLA_2_) show synergistic action with lipid membranes, leading to cell damage [58].

### 3.1. Allergic Reactions

Melittin is considered as an allergen of bee venom since research in the 1970s showed that it induces an IgE response in around one-third of honeybee venom-sensitive patients [59] and melittin appears to be allergenic in several patients [60]. However, the results are possibly due to contamination with other bee venom components. Bee venom is a complex mixture of melittin, apamine, mast cell degranulating peptide, histamine, adolapin, oligopeptides, phospholipids, saccharides, phospholipase A_2_, hyaluconidase, acid phosphatase, *etc.* [26]. Purified melittin potentially contains residues of strong allergens such as hyaluronidase, phospholipase A_2_ and acid phosphatasen. As the purity of currently available purified melittin is not high (e.g., Sigma-Aldrich M7391: 65%–85% and Sigma-Aldrich M2272: ≥85%, (Sigma Aldrich Inc., St. Louis, MO, USA)) synthesized melittin (e.g., Sigma-Aldrich M4171: ≥97%) should be used to confirm whether melittin causes allergic reactions or not.

### 3.2. Hemolysis, Cytotoxic Effects

Melittin is also known for its high lytic activity on human erythrocyte cells [61,62]. It directly binds on erythrocytes and releases hemoglobin. It is reported that the maximum capacity and the apparent dissociation constant for melittin binding to human erythrocytes is 1.8 × 10^7^ molecules/cell and 30 nM, respectively [61]. When melittin provokes hemolysis, swelling of the erythrocytes is observed after leakage of cations occurs from membrane of the cells. In its initial phase, melittin increases permeability of ions and the release of hemoglobin is followed [61,62]. Melittin is cytotoxic for human peripheral blood lymphocytes (HPBLs) in a dose- and time-dependent manner. It leads to granulation, morphologic changes, and finally lysis of cells [63].

### 3.3. Genotoxic Effects

Gajski *et al.* report that low doses (non-cytotoxic concentrations) of melittin can increase DNA damage in HPBLs [63]. In their experiments, comet and micronucleus assays show decreased proliferation of lymphocyte and increased formation of nuclear buds and micronuclei. Moreover, melittin modulates gene expressions related with apoptosis (Bax, Bcl-2, Cas-3, Cas-7), DNA damage response (TP53, CDKN1A, GADD45α, MDM), and oxidative stress (CAT, GCLC, GPX1, GSR, SOD1). The observed genotoxicity coincides with reduction of glutathione level, increased formation of reactive oxygen species, increased phospholipase C activity and lipid peroxidation, showing the induction of oxidative stress.

## 4. Attempts to Overcome the Adverse Effect of Melittin

Even though melittin has demonstrated significant therapeutic properties, its toxicity must be neutralized for use as an anti-inflammatory agent. The cytotoxic effect of melittin is beneficial in part for anti-tumor purposes, but it hinders therapeutic application of melittin for other purposes. Since the native form of melittin causes non-specific cell lysis and toxicity, attempts including mutation and fusion proteins to reduce the toxicity of melittin to deliver it to specific targeted lesions are ongoing. Asthana *et al.* showed that alanine substitution in the leucine zipper motif of melittin results in a significant reduction of its hemolytic, but not anti-bacterial activity [64]. This suggests that lesion- specific mutations can reduce the toxicity of melittin without affecting its therapeutic activity. Rayahin *et al.* showed that the melittin fusion protein with glutathione S-transferase exhibits anti-inflammatory properties and minimal toxicity [65]. Development of delivery techniques using nanocarriers enable safe delivery of melittin to targeted lesions without affecting non-target cells [27,66,67].

## 5. Conclusions

Studies over the previous decade have advanced our knowledge about bee venom-derived melittin. Herein, we summarize the inflammation-specific effects of melittin and discuss its underlying mechanisms (Table 1, Figure 1). However, parts of the underlying mechanisms still remain unclear and the toxicity of this peptide still prevents its use for anti-inflammatory activity. Melittin could possibly be used for anti-inflammatory purposes if careful provisions are taken to avoid adverse effects. Technical developments will help to modify this toxic peptide into a safe therapeutic agent. We consider that synthesized melittin and its derivatives [68,69,70] should be used to overcome the effects of contaminants from bee venom and for developing novel pharmaceutical agents. The future therapeutic application of melittin on inflammatory disorders will depend on new study protocols to validate the efficiency and safety of melittin. In addition, the anti-inflammatory effect of bee venom PLA_2_ was recently established [71]. As melittin and bee venom PLA_2_ are synergistic [58,72], it is worth studying the anti-inflammatory effect of melittin and bee venom PLA_2_ co-treatment.

## Figures and Tables

**Table 1:** Anti-inflammatory effects of melittin.

Disease Model	Specific Effects	Experimental System	Dose	Reference
Acne vulgaris	Reduced IKK, IκB, NF-κB and p38 phosphorylation	HaCaT cells, *in vitro*	0.1–1 µg/mL	[37]
	Reduced swelling and granulomatous responses	mouse, *in vivo*, melittin and vaseline mixture applied to the surface of ear	1–100 µg/ear	
	Suppressed TLR2 and CD14			
	Inhibited mRNA expression of TNF-α, IL-1β, IL-8, and IFN-γ.			
	Decreased expression of TNF-α and IL-1β by regulation of TLR2 and 4	human THP-1 monocytic cell, *in vitro*	0.1–1 µg/mL	[38]
	Inhibited apoptosis and cleavage of caspase-3, -8, and PARP			
Neuro inflammtion	Suppressed NO and iNOS expression	BV2 microglia, *in vitro*	0.5–2 µg/mL	[39]
	Suppressed NF-κB activation by blocking degradation of IκBα and phosphorylation JNK and Akt			
	Suppressed expression IL-1β, IL-6, TNF-α, PGE2			
	Increased cell viability and decrease apoptosis	SH-SY5Y cells, *in vitro*	0.5–2 µg/mL	[40]
Amyotrophic lateral sclerosis	Decreased number of microglia and phospo-p38 in the spinal cord and brainstem	mouse, *in vivo*, s.c. injection at ST36 acupoint twice a week	0.1 µg/g	[41]
	Improved motor function and inhibit neuronal death in the spinal cord			
	Inhibited a-synuclein misfolding			
	Suppressed expression of Iba-1 and CD14 in the lung	mouse, *in vivo*, s.c. injection at ST36 acupoint three times a week	0.1 µg/g	[42]
	Suppressed expression of CD14 and COX-2 in spleen			
	Increased pERK and Bcl-2 in spleen			
Atherosclerosis	Inhibited PDGR β-tyrosine phosphorylation and its intracellular signal transduction	rat aortic vascular smooth muscle cell, *in vitro*	0.4–0.8 µg/mL	[43,44]
	Decreased total cholesterol and triglyceride but increased HDL in serum	mouse, *in vivo*, i.p. injection, twice a week	0.1 mg/kg	[45]
	Decreased expression of TNF-α, IL-1β, VCAM-1, ICAM-1, and TGF-β1			
	Inhibited expression IL-1β, TNF-α and NF-κB activation	human monocytic cell line THP-1 derived macrophages, *in vitro*	0.1–1 µg/mL	
	Increased prohibitin, annexin-1 expression	TNF-α stimulated human vascular smooth muscle cells, *in vitro*	2 µg/mL	[46]
	Inhibited calreticulin expression reduced the phosphorylation of EGFR, and ERK and the expression of NF-κB in nuclear			
Arthritis	Inhibited expression of LPS-induced COX-2, PGE2, cPLA2, NO and iNOS	raw 264.7 and synoviocytes obtained from patients with rheumatoid arthritis, *in vitro*	5–10 µg/mL	[20,47,48]
	Inhibited JUK and NF-κB activation, release of IκB, and nuclear translocation of the p50 subunit			
Liver inflammation	Suppressed inflammation, fibrosis, and expression of VCAM-1, IL-6 and TNF-α in the liver	mouse, *in vivo*, i.p. injection, twice a week for 12 weeks	0.1 mg/kg	[49]
	Suppressed expression of IL-1β, IL-6 and TNF-α	rat primary hepatic stellate cells, *in vitro*	0.1–1 µg/mL	
	Suppressed apoptosis and TNF-α, IL-1β and NF-κB signaling in GalN/LPS induced acute hepatic failure	mouse, *in vivo*, i.p. injection	0.1 mg/kg	[50]
	Suppressed apoptotic pathway and NF-κB activation	mouse hepatocyte cell lines AML12	0.5–2 µg/mL	[51]
	Suppressed expressions of TNF-α, IL-6 and p-STAT3 in chronic liver injury	mouse, *in vivo*, i.p. injection, twice a week for 4 weeks	0.1 mg/kg	[52]
